# Effects of Plyometric Jump Training on Physical Fitness in Amateur and Professional Volleyball: A Meta-Analysis

**DOI:** 10.3389/fphys.2021.636140

**Published:** 2021-02-26

**Authors:** Rodrigo Ramirez-Campillo, Antonio García-de-Alcaraz, Helmi Chaabene, Jason Moran, Yassine Negra, Urs Granacher

**Affiliations:** ^1^Human Performance Laboratory, Department of Physical Activity Sciences, Universidad de Los Lagos, Osorno, Chile; ^2^Facultad de Ciencias, Centro de Investigación en Fisiología del Ejercicio, Universidad Mayor, Santiago, Chile; ^3^Faculty of Educational Sciences, University of Almeria, Almería, Spain; ^4^LFE Research Group, Universidad Politécnica de Madrid, Madrid, Spain; ^5^Division of Training and Movement Sciences, University of Potsdam, Potsdam, Germany; ^6^High Institute of Sports and Physical Education, Kef, University of Jendouba, Jendouba, Tunisia; ^7^School of Sport, Rehabilitation and Exercise Sciences, University of Essex, Colchester, United Kingdom; ^8^Research Unit (UR17JS01) ≪Sport Performance, Health & Society≫, Higher Institute of Sport and Physical Education of Ksar Saîd, University of “La Manouba”, Manouba, Tunisia

**Keywords:** human physical conditioning, athletic performance, resistance training, stretch-shortening cycle, exercise, team sports

## Abstract

We aimed to examine the effects of plyometric jump training (PJT) on measures of physical fitness in amateur and professional volleyball players. A systematic electronic literature search was carried out in the databases PubMed, MEDLINE, Web of Science, and SCOPUS. Controlled studies including pre-to-post intervention tests of physical fitness and involving healthy volleyball players regardless of age and sex were considered. A random-effects model was used to calculate effect sizes (ES) between intervention and control groups. Moderator analyses considered programme duration, training frequency, total number of training sessions and jumps, participants' sex, age, and expertise level. The Physiotherapy Evidence Database scale was used to assess the methodological quality of the included studies. Eighteen moderate-to-high quality (median of 5 PEDro points) studies were eligible, comprising a total of 746 athletes. None of the included studies reported injuries related to the PJT intervention. The main findings showed small-to-moderate effects (*p* < 0.05) of PJT on linear sprint speed (ES = 0.70), squat jump (ES = 0.56), countermovement jump (CMJ) (ES = 0.80), CMJ with arm swing (ES = 0.63), drop jump (ES = 0.81), and spike jump height (ES = 0.84). Sub-analyses of moderator factors included 48 data sets. Only age had a significant effect on CMJ performance. Participants aged ≥16 years achieved greater improvements in CMJ performance compared to <16 years old (ES = 1.28 and 0.38, respectively; *p* = 0.022). No significant differences (*p* = 0.422) were identified between amateur (ES = 0.62) and professional volleyball players (ES = 1.01). In conclusion, PJT seems safe and is effective in improving measures of physical fitness in amateur and professional volleyball players, considering studies performed in both male and female.

## Introduction

Plyometric jump training (PJT) is one of the most popular training approaches adopted by coaches and strength and conditioning professionals in both, team and individual sports (Ebben and Blackard, 2001; Ebben et al., 2004, 2005; Blagrove et al., 2017). The importance of PJT has principally been gained from the increasing number of scientific studies demonstrating its effectiveness in improving a wide range of physical fitness components (e.g., muscle power, linear sprint, and change-of-direction speed), irrespective of age, sex, and training expertise (de Villarreal et al., 2009). Generally, PJT benefits from the mechanical properties of the stretch-shortening cycle (SSC) during jump drills (Komi and Gollhofer, 1997; Taube et al., 2012). Based on the time spent on the ground, jump drills can be classified as fast SSC (i.e., short ground contact time; <250 ms) or slow SSC (i.e., long ground contact time; >250 ms) exercises (Duda, 1988; Sands et al., 2012; Faigenbaum and Chu, 2017). PJT has been shown to enhance neuromuscular (e.g., improved neural drive to agonist's muscles) and/or mechanical/structural properties (e.g., alterations to musculotendinous stiffness and architecture) (Markovic and Mikulic, 2010). These positive effects on neuromuscular and structural properties should have potential in sports such as volleyball, which involves extensive movements analogous to PJT drills.

Volleyball is a team sport characterized by intermittent efforts with periods of short duration (i.e., 3–9 s), high-intensity activities, interspersed with relatively long periods (i.e., 10–20 s) of recovery (Polglaze and Dawson, 1992). Earlier studies analyzing competition performance have shown that accelerations, decelerations, jumping, ball-striking, and multidirectional locomotions are common movements in volleyball (Sheppard et al., 2007; García de Alcaraz et al., 2017; Garcia-de-Alcaraz et al., 2020). Indeed, although total sprint distance in volleyball players may be lower compared to other team sports (e.g., soccer) (Stolen et al., 2005), a relatively greater portion (i.e., ~30%) of total movement distance in volleyball is performed while sprinting, particularly linear sprinting (Polglaze and Dawson, 1992). In addition, a quick running approach before the jump is also related to a better jump height (Wagner et al., 2009) and greater linear sprinting speed is specked in professional compared to amateur volleyball players (Smith et al., 1992). Moreover, point-scoring actions (e.g., serve, spike, and block) are jump-based, with a typical squad (n~12) of volleyball players performing ~120,000 jumps throughout a season (Garcia-de-Alcaraz et al., 2020). According to the principle of training specificity, volleyball players should systematically engage in jump-based exercise programs.

Indeed, some investigations have demonstrated the beneficial effects of PJT on measures of physical fitness in amateur and professional volleyball players (Martel et al., 2005; Ziv and Lidor, 2010; Behrens et al., 2014). However, a recent scoping review including 420 studies indicated that research exploring the effects of PJT is generally limited by the small size of the recruited samples, with 70% of volleyball studies including 12 participants, or fewer, per group (Ramirez-Campillo et al., 2020c). Indeed, even if significant effects are observed in small samples, it may not be possible to verify the legitimacy of a result due to considerable overestimation of the population effect size, the probability of low precision in the population estimate, and reduced replicability (Abt et al., 2020). One of the effective approaches to solve the problem of underpowered studies is to conduct a meta-analysis by aggregating data from earlier studies to increase total statistical power (Liberati et al., 2009). This approach facilitates the drawing of stronger inferences about the effectiveness of PJT.

A number of reviews and meta-analyses addressing the effects of PJT on components of physical fitness have been published (Saez de Villarreal et al., 2012; Asadi et al., 2016; Stojanović et al., 2017). However, these meta-analyses incorporated participants across a range of different sports. Because the effects of PJT may vary depending on the sports background of the athlete, findings from these studies cannot be generalized. For example, PJT-related improvements in change-of direction speed were four-fold larger in basketball compared with rugby players (Asadi et al., 2016). Moreover, strength improvements following PJT have been reported to be twice as high in volleyball compared to basketball players (de Villarreal et al., 2010). Moreover, differences were found in terms of jump landing biomechanics in response to an injury prevention program that included PJT in female basketball compared with soccer players (Taylor et al., 2018). PJT-related differences were observed for bone-related anthropometric indices, linear sprint, and vertical jump performances (i.e., significant improvements in swimmers and cyclists but not in soccer players) after 36 weeks of PJT (Vlachopoulos et al., 2018). Finally, PJT induced improvements in linear sprint performance in basketball players (ES = 0.68) but not in volleyball players (ES = −0.12) (de Villarreal et al., 2012). Moreover, to the best of our knowledge, there are no meta-analyses that addressed the effects of PJT on a wide range of physical fitness measures (e.g., sprinting speed, sport-specific jump performances, reactive strength) in volleyball players. The only available meta-analysis (Ramirez-Campillo et al., 2020b) aggregated data on the effects of PJT on solely muscle power (countermovement jump [CMJ]) in volleyball players. Although CMJ is a relevant measure of lower-limbs muscle power for volleyball players, sport-specific jump actions in volleyball have not been considered in the previous meta-analysis. Different types of jumps (e.g., spike jump; CMJ with arms; block displacement plus jump), in addition to other actions such as sprinting (e.g., spike running approach), are crucial during competitive matches (Sheppard et al., 2007; García de Alcaraz et al., 2017; Garcia-de-Alcaraz et al., 2020) and are likely to be a more appropriate reflection on volleyball performance than the generic CMJ. Accordingly, a meta-analysis seeking to examine the effects of PJT on those measures of physical fitness is needed.

Given the increased scientific awareness on the effectiveness of PJT (Ramirez-Campillo et al., 2018a, 2020c) and the lack of any meta-analyses focusing on the effects of PJT on broader measures of physical fitness, this meta-analysis sought to examine the effects of PJT on a wide range of measures of physical fitness (i.e., squat jump; spike jump; countermovement jump with arms; sprinting) relevant to amateur and professional volleyball players. Based on the outcomes of previous studies (Silva et al., 2019), it was hypothesized that PJT is effective to improve jump (i.e., squat jump; countermovement jump; drop jump; spike jump; countermovement jump with arms) and sprint performances (linear sprint speed for 10-m up to 50-m distance) in volleyball players. The aim of this systematic review with meta-analysis was to examine the effects of PJT vs. controls on components of physical fitness in amateur and professional volleyball players. In addition, we were interested in the role of potential moderating factors such as PJT duration (number of weeks), frequency (number of sessions per week), total number of training sessions, and the total number of jumps. In various sub-analyses, we studied the effects of participants' sex, age, and expertise level (i.e., professional and amateur) on the overall outcomes. Finally, we aimed at elucidating whether single-mode PJT has different effects compared with multimodal training in which PJT constitutes one element.

## Methods

This meta-analysis was conducted following the guidelines of the Preferred Reporting Items for Systematic Reviews and Meta-Analyses (PRISMA) statement (Liberati et al., 2009).

### Eligibility Criteria

A PICOS (participants, intervention, comparators, study outcomes, and study design) approach was used to rate studies for eligibility. The inclusion/exclusion criteria were defined *a priori* as follows: (1) population: cohorts of healthy volleyball players with no restriction for age or sex, irrespective of the expertise status; (2) intervention: a PJT programme of at least 2 weeks duration which included lower-body jumping, bounding, or hopping actions that commonly utilize a pre-stretch or countermovement that solicits the SSC (Chu and Myer, 2013; Moran et al., 2018a; Ramirez-Campillo et al., 2018a); (3) comparator: an active control group of healthy volleyball players without current involvement in PJT; (4) study outcomes: a measure of physical fitness (e.g., jump, sprint speed); and (5) study design: controlled trials.

Exclusion criteria involved: (i) studies that mixed volleyball players with athletes from other sports; (ii) studies that did not incorporate any measure of physical fitness; (iii) studies that did not include an active comparator, (iv) studies written in a language other than English. Only articles published in the English language were considered given potential difficulties to translate articles written in different languages, and the fact that 99.6% of PJT literature is published in English (Ramirez-Campillo et al., 2018a); (v) studies that did not include a pre-to-post intervention assessment of at least one physical fitness outcome; (vi) non-peer-reviewed articles, although gray literature sources (e.g., conference proceedings) were also considered if a full-text version was available; (vii) abstracts, case reports, cross-sectional, reviews, or a training-related study that did not focus on the effects of PJT exercises; and (viii) retrospective/prospective studies, studies in which the use of jump exercises was not clearly described, special communications, repeated-bout effect interventions, repeated references, letters to the editor, invited commentaries, errata, overtraining studies, and detraining studies. Regarding detraining studies, if there was a training before a detraining period, the study was considered for inclusion, ignoring the detraining period in the analysis.

### Information Sources

The electronic databases PubMed, MEDLINE, Web of Science, and SCOPUS were systematically searched for relevant studies. Keywords were collected through experts' opinion, a systematic literature review, and controlled vocabulary (e.g., Medical Subject Headings: MeSH). A boolean search syntax using the operators “AND” and “OR” was applied. The words “ballistic,” “complex,” “explosive,” “force-velocity,” “plyometric,” “stretch-shortening cycle,” “jump,” “training,” and “volleyball” were used. The following is an example of a PubMed search: (((((((((“randomized controlled trial”[Publication Type]) OR “controlled clinical trial”[Publication Type]) OR “randomized”[Title/Abstract]) OR “trial”[Title]) OR “clinical trials as topic”[MeSH Major Topic]) AND “volleyball”[Title/Abstract]) OR “volleyball players”[Title/Abstract]) OR “volleyball/physiology”[Title/Abstract]) AND “training”[Title/Abstract]) OR “plyometric”[Title/Abstract]. After an initial search, accounts were created in the respective databases. Through these accounts, the lead investigator received automatically generated emails for updates regarding the search terms used. These updates were received daily (if available), from December 2019, and studies were eligible for inclusion until July 31, 2020. Following the formal systematic searches, additional hand-searches were conducted.

### Study Selection

When selecting studies for inclusion, retrieved articles were first screened for duplicates through specialized software (EndNote X8 for Windows, Clarivate Analytics). After duplicates removal, a review of all relevant article titles was conducted before an examination of article abstracts and then full-published articles. Two authors (RRC-AGA) conducted the process independently. Potential discrepancies between the two reviewers were resolved by consensus. Full-text articles excluded, with reasons, were recorded.

### Data Collection Process

Data were extracted from gathered articles, using a custom-made Microsoft Excel matrix data (Microsoft Corporation, Redmond, WA, USA).

### Data Items

Physical fitness measures included speed (linear sprint speed) and proxies of muscle power (jumping) and were selected as main/primary outcomes of this meta-analysis. The testing procedures for linear sprint speed involved maximal-effort running attempts through distances from 10-m up to 50-m. Moreover, the jumping height testing procedures involved maximal single-effort bilateral vertical jumps, including squat jump, countermovement jump, drop jump, spike jump, and countermovement jump with arm swing. Adverse health effects, including soreness, pain, fatigue, injury, and damage, were chosen as the secondary outcomes.

Extracted data also included the following information: quality of PJT treatment description, type of control, type of randomization, and number of participants per group. In addition, participants' sex, age (years), body mass (kg), height (m), previous experience with PJT (yes/no, i.e., as described in studies), and expertise level (e.g., professional/amateur) were extracted. Regarding PJT characteristics, extracted data also included the frequency of training (days/week), duration (weeks), intensity level (e.g., maximal/moderate), and marker of intensity (e.g., jumping height), jump box height (cm), number of total jumps completed during the intervention, types of jump drills performed, the combination (if applicable) of PJT with another form of training type, rest time between sets (s), rest time between repetitions (s), rest time between sessions (hours), type of jumping surface (e.g., grass), type of progressive PJT overload (e.g., volume-based; technique-based), training period of the year (e.g., in-season), replaced (if applicable) portion of the regular training with PJT, and tapering strategy (if applicable). A complete description of the aforementioned PJT characteristics has been previously published (Ramirez-Campillo et al., 2018a, 2020c).

### Methodological Quality of Included Studies

The Physiotherapy Evidence Database (PEDro) scale (Maher et al., 2003; de Morton, 2009) was used to assess the methodological quality of the included studies. This scale evaluates different aspects of the study design, such as participant eligibility criteria, randomization, blinding, attrition, and reporting of data. There are 11 items included in the PEDro checklist, but item 1 is not rated. Therefore, the minimum possible score on the checklist is 0 and the maximum 10. As in a similar previous PJT meta-analysis (Stojanović et al., 2017), the quality assessment was interpreted as follows: ≤ 3 points was considered poor quality, 4–5 points as moderate quality, and 6–10 points as high quality. If trials had already been assessed and listed on the PEDro database (or similar sources), such scores were adopted. Two reviewers (RRC and AG) performed the methodological quality assessment independently. Disagreements between reviewers rating were resolved through discussion and consensus with a third author (YN).

### Summary Measures

Meta-analyses were conducted when at least three studies provided data for physical fitness outcomes (Moran et al., 2018a; Garcia-Hermoso et al., 2019; Skrede et al., 2019). Means and standard deviations (SD) for a measure of pre-post-intervention performance were used to calculate between-group effect sizes (ES; Hedge's *g*). The data were standardized using post score SD. If authors did not provide sufficient data (missing or in graphics), the corresponding author was contacted and we kindly asked for the respective information (Newton et al., 1999; Maffiuletti et al., 2002; Martel et al., 2005; Kamalakkannan et al., 2011; Behrens et al., 2014; Pereira et al., 2015; Usman and Shenoy, 2015, 2019; Çımenlı et al., 2016; Turgut et al., 2016; Gjinovci et al., 2017; Amato et al., 2018; Idrizovic et al., 2018). If authors did not respond to our query or authors could not provide the requested data, the study outcome was excluded from the analysis. However, if data were displayed in a figure and no numerical data were provided in the text or tables and authors did not respond to our queries, we used a validated software tool (*r* = 0.99, *p* < 0.001) (Drevon et al., 2016) (WebPlotDigitizer; https://apps.automeris.io/wpd/) to derive means and standard deviations from figures. Therefore, no articles were excluded *a priori* if authors did not provide sufficient information.

The inverse-variance random-effects model for meta-analyses was used because it allocates a proportionate weight to trials based on the size of their individual standard errors (Deeks et al., 2008) and facilitates analysis while accounting for heterogeneity across studies (Kontopantelis et al., 2013). This approach was used to better account for the inaccuracy in the estimate of between-study variance (Hardy and Thompson, 1996). The effect size (ES) are presented alongside 95% confidence intervals (CIs), and were interpreted as follows: <0.2, trivial; 0.2–0.6, small; >0.6–1.2, moderate; >1.2–2.0, large; >2.0–4.0, very large; >4.0, extremely large (Hopkins et al., 2009). In cases in which there were more than one intervention group (i.e., studies with more than one intervention arm and one control arm), the control group was proportionately divided to facilitate comparison across all participants (Higgins et al., 2008). All analyses were carried out using the Comprehensive Meta-Analysis programme (version 2; Biostat, Englewood, NJ, USA).

### Synthesis of Results and Risk of Bias Across Studies

Heterogeneity was assessed using the *I*^2^ statistic. Values of <25, 25–75, and >75%, were considered to represent low, moderate, and high levels of heterogeneity, respectively (Higgins et al., 2003). The risk of bias across studies was assessed using the extended Egger's test (Egger et al., 1997). In the case of a significant Egger's test, a sensitivity analysis was performed.

### Additional Analyses

To assess the potential effects of moderator variables, subgroup analyses were performed. Using a random-effects model, potential sources of heterogeneity likely to influence the effects of training were selected *a priori*: programme duration (number of weeks), training frequency (number of sessions per week), total number of training sessions, and the total number of jumps (Moran et al., 2018b, 2019). The sex, the age (≥16 years old compared to <16 years old), and the expertise level of the participants were also considered as moderator variables. Participants were divided using a median split (Moran et al., 2017b, 2018b, 2019). Regarding the expertise level of the athletes, studies were divided into those that incorporated professional (i.e., those participating in national and international tournaments) and amateur athletes (e.g., those participating in local competitions, or recreationally active). *A posteriori*, an additional analysis was performed for studies that applied single-mode PJT compared with studies that conducted PJT in combination with another training type (e.g., resistance training). Meta-analyses stratification by each of these factors was performed, with a *p* < 0.05 considered as the threshold for statistical significance.

## Results

### Study Selection

**Figure 1:**
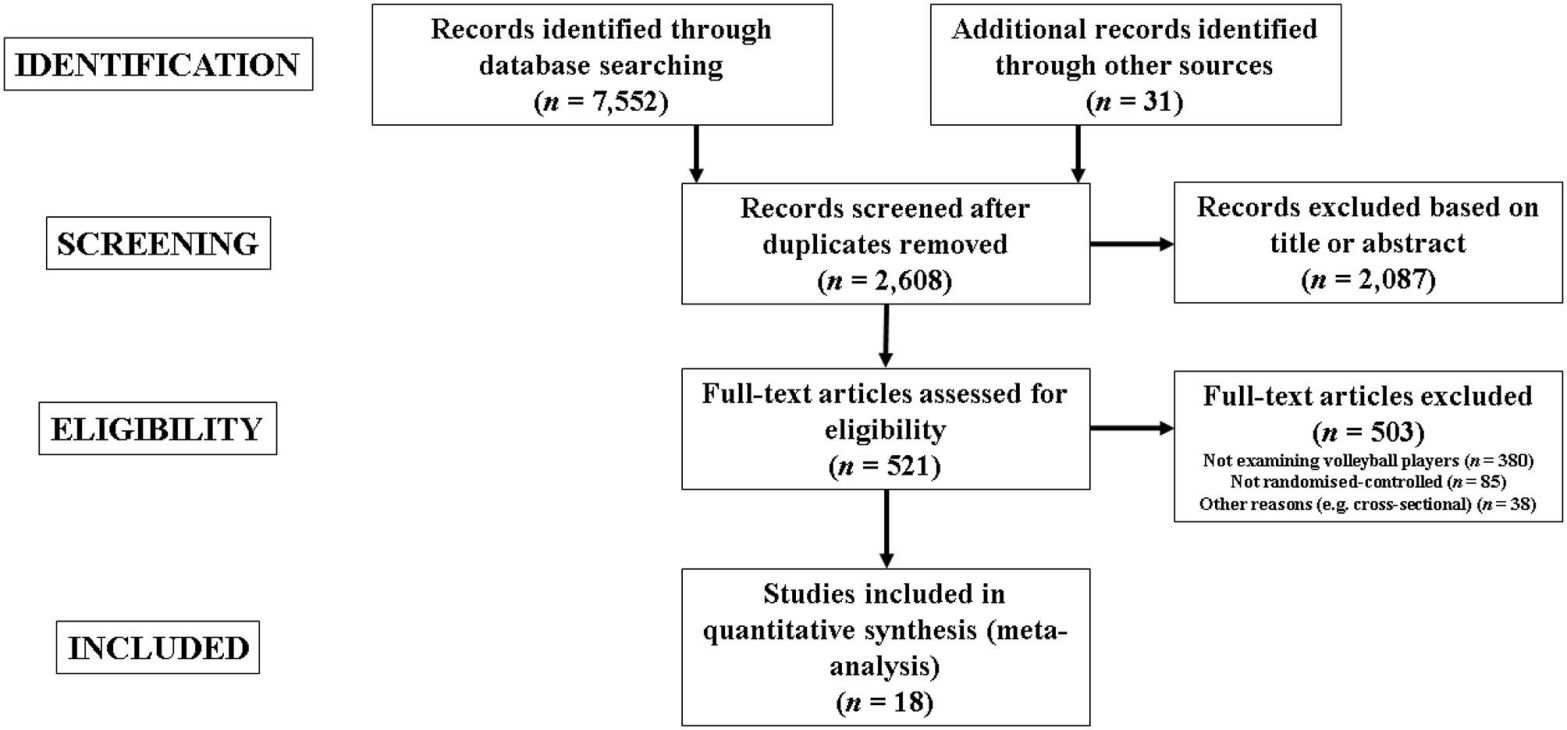
Flow diagram of the search process.

Figure 1 provides a graphical schematisation of the study selection process. Through database searching, 7,552 records (2,373 from PubMed, 2,396 from SCOPUS, and 2,783 from WOS) were initially identified, and another 31 records were added from other sources (manual search in the author's libraries). From these, duplicates were removed (*n* = 4,975), with 2,608 records remaining. Study titles were screened with 1,278 studies being removed. After this, article abstracts were screened for relevance with 809 studies being removed, and 521 full-text articles remained and were assessed for eligibility. After applying all inclusion/exclusion criteria, 18 controlled trials were eligible for meta-analysis (Newton et al., 1999; Maffiuletti et al., 2002; Martel et al., 2005; Kamalakkannan et al., 2011; Behrens et al., 2014; Pereira et al., 2015; Usman and Shenoy, 2015, 2019; Çımenlı et al., 2016; Kristicevic et al., 2016; Trajkovic et al., 2016; Turgut et al., 2016; Gjinovci et al., 2017; Amato et al., 2018; Idrizovic et al., 2018; Fathi et al., 2019; Ho et al., 2019; Wang et al., 2020). These studies comprised 24 separate experimental groups and 18 control groups. Most control groups continued with their regular volleyball training (Maffiuletti et al., 2002; Pereira et al., 2015; Usman and Shenoy, 2015, 2019; Kristicevic et al., 2016; Trajkovic et al., 2016; Amato et al., 2018; Idrizovic et al., 2018; Fathi et al., 2019; Ho et al., 2019; Wang et al., 2020). Four studies (Newton et al., 1999; Martel et al., 2005; Turgut et al., 2016; Gjinovci et al., 2017) included active controls (e.g., additional volleyball skill-based training; flexibility drills; slow speed resistance training drills), one study (Behrens et al., 2014) did not report specific information on controls and two studies (Kamalakkannan et al., 2011; Çımenlı et al., 2016) reported passive control groups. Of note, studies that incorporated experimental groups that combined PJT with other training modalities were not excluded as long as the PJT load represented a meaningful (e.g., ≥50% of exercises per session) portion of the intervention. In this sense, the study of Amato et al. (Amato et al., 2018) was not excluded as two of three exercises involved jump-landing tasks. Similarly, the study of Fathi et al. (Fathi et al., 2019) involved two experimental groups, one performed single-mode PJT, and the other combined PJT with resistance exercises. Moreover, the study from Usman and Shenoy (Usman and Shenoy, 2019) involved two experimental groups, one performed single-mode PJT while the other conducted combined PJT exercises (*n* = 8) together with dynamic stretching (*n* = 5). In addition, the study of Maffiuletti et al. (Maffiuletti et al., 2002) involved one experimental group that combined performed PJT with electrostimulation (50 of the 128 maximal contractions involved jumps).

### Study Characteristics

The characteristics of PJT intervention programmes and participants are displayed in Tables 1, 2, respectively. The included studies comprised 746 volleyball players eligible. The participants' mean age across the eligible studies extends from 11.6 to 24.0 years. The duration of PJT interventions ranges from 4 to 16 weeks, with training frequency ranges from one to three sessions per week.

**Table 1:** Characteristics of included participants.

**References**	***n***	**Gender**	**A**	**BM**	**H**	**SPT**	Expertise level^*^
Amato et al. (2018)	12	F	11.6	48.5	156	NR	Normal to moderate/U14
Behrens et al. (2014)	13	M-F	24	77	183	No	Normal to moderate/Senior (no-professional)
Çımenlı et al. (2016)	12 (wood)	M	21.0	73.7	184	NR	Moderate to high/Senior (no-professional)
	12 (synthetic)			83.1	185		
Fathi et al. (2019)	20 (RT)	M	14.7	68.7	177	No	Moderate to high/U16
	20		14.6	67.9	178		
Gjinovci et al. (2017)	21	F	21.9	60.8	176	Yes	High/Senior (professional)
Ho et al. (2019)	12	M	20.2	76.5	182	NR	High (professional)
Idrizovic et al. (2018)	13	F	16.6	59.4	175	NR	High/U18
Kamalakkannan et al. (2011)	12 (water, with weights)	NR	19.0	NR	NR	NR	Normal to moderate/Varsity
	12 (water)						
Kristicevic et al. (2016)	28	F	15.4	60.8	171	NR	Moderate to high/U16
Maffiuletti et al. (2002)	10	M	21.8	80.5	191	NR	Moderate/Senior (no-professional)
Martel et al. (2005)	10	F	15.0	64.0	167	No	High/U16
Newton et al. (1999)	8	M	19.0	84.0	189	Yes	High/Varsity
Pereira et al. (2015)	10	F	14.0	52.0	160	No	Moderate/U14
Trajkovic et al. (2016)	32	F	17.2	64.8	176	NR	Moderate to high/U18
Turgut et al. (2016)	8 (weighted jump rope)	F	15.0	59.4	166	NR	Moderate to high/U16
	9 (standard jump rope)		14.1	57.7	165		
Usman and Shenoy (2019)	30	M	19.6	66.0	176	Yes	Low to high/Varsity
	30 (stretching)						
Usman and Shenoy (2015)	30	M	19.2	66.0	176	No	Low to high/Varsity
	30	F					
Wang et al. 2020	10	M	21.5	78.1	187	NR	High (professional)

A, age of subject (years); BM, body mass (kg); F, female; H, height of participants (cm); M, male; N, number of participants. The information provided in parenthesis denote studies that included two intervention arms with different characteristics, indicating (i) the type of surface used during PJT (i.e., wood; synthetic floor; water; water while carrying additional loads), (ii) if the PJT was combined with an additional type of training (i.e., RT; stretching), (iii) additional equipment used during PJT (i.e., jump rope; weighted jump rope); NR, non-reported; PJT, plyometric jump training; RT, resistance training; SPT, systematic plyometric jump training experience; U14, U16, U18, under 14, 16, 18 years of age competitive categories;

* *Expertise level: high, for professional/elite athletes with regular enrolment in national and/or international competitions, highly trained participants with >10 training hours per week or >6 training sessions per week and scheduled official and friendly competitions. Moderate, for non-elite/professional athletes, with a regular attendance in regional and/or national competitions, between 5 and 9.9 training hours per week or 3–5 training sessions per week and scheduled official and friendly competitions. Normal, for recreational athletes with <5 training hours per week with sporadic competitions' participation, and for physically active participants and school-age youths regularly involved in physical education classes*.

**Table 2:** Characteristics of PJT programs.

**References**	**Freq**	**Wk**	**Int**	**BH**	**TJ**	**Type**	**Comb**	**RBSE**	**RBR**	**RBTS**	**Surf**	**PO**	**TP**	**R**	**T**
Amato et al. (2018)	2	6	NR	40	880	Mix	Isometric squat	90	NR	NR	NR	V	NR	NR	No
Behrens et al. (2014)	2	8	Maximal	40	972	Mix	No	90	4	NR	Rigid	V	IS	No	No
Çımenlı et al. (2016)	3	8	NR	30–70	3,000	Mix	No	120	NR	NR	Wood	T, V	PS	NR	No
											Synthetic				
Fathi et al. (2019)	2	16	Low, moderate and high^*^	30–50	576	Mix	RT	90	5-10	NR	NR	V, T, I	IS	NR	No
				30–40	1,184		No								
Gjinovci et al. (2017)	2	12	Low, moderate and high^*^	NR	>924	Mix	No	120 - 240	NR	NR	NR	I, V	NR	No	No
Ho et al. (2019)	3	6	High, moderate^*^	20–76	2,082	Mix	No	NR	NR	NR	NR	T	NR	Yes	Yes
Idrizovic et al. (2018)	1	12	Low, moderate and high^*^	20–60	2,484	Mix	No	120–300	NR	168	Wood	V, T, I	PS	No	No
Kamalakkannan et al. (2011)	3	12	NR	NR	4,080	Mix	No	30 - 90	NR	NR	Water	V, T	NR	NR	Yes
Kristicevic et al. (2016)	2	5	Maximal	20–40	920 + 1,260 s	Mix	No	NR	NR	48–120	NR	V, I	IS	Yes	Yes
Maffiuletti et al. (2002)	3	4	Maximal	40	600	RBVJ	Electro stimulation	180	NA	NR	NR	No	PS	No	No
Martel et al. (2005)	2	6	Maximal	61	>138	Mix	No	30	NR	NR	Water	V	PS	No	No
Newton et al. (1999)	2	8	30–80% 1RM	NA	576	Loaded jump squat	No	NR	NR	NR	NR	I	PS	Yes	No
Pereira et al. (2015)	2	8	Maximal	NA	2,376	Mix	No	120–180	NR	48	NR	V, I	IS	No	No
Trajkovic et al. (2016)	2	6	NR	20–60	1,278 + 2,100 s	Mix	No	NR	NR	NR	NR	V, I	PS-IS	NR	No
Turgut et al. (2016)	3	12	NR	NA	5,490 s	Rope jumps	No	30–60	NA	NR	NR	V	NR	Yes	No
Usman and Shenoy (2019)	2	8	Low to high^*^	30–80	2,976	Mix	No	60–600	5–10	NR	NR	I	NR	NR	No
							Stretching								
Usman and Shenoy (2015)	2	8	Low to high^*^	30–80	2,976	Mix	No	60–300	5–10	48–120	NR	I	NR	NR	No
Wang et al. (2020)	2	6	High, moderate^*^	20–76	1,388	Mix	No	NR	NR	NR	NR	I	NR	NR	Yes

BH, box height for plyometric drop jumps (cm); Comb, combined; Freq, frequency of PJT (days/week); Int, intensity of PJT. In terms of maximal, this involved either maximal absolute effort to achieve maximal height, distance, reactive strength index, velocity, or another marker of intensity. As to the study of Newton et al. (1999), a relative intensity of 30–80% of the one repetition maximum (1RM) was used. For the studies marked with an

* *the intensity was reported only qualitatively; IS, in-season; NA, non-applicable; NR, non-reported; PJT, plyometric jump training; PO, progressive overload, in the form of either volume (i.e., V), intensity (i.e., I), type of drill (i.e., T), or a combination of these; PS, pre-season; R, replacement of a portion of the habitual training drills with plyometric jump training drills; RBR, rest between repetitions (seconds); RBSE, rest between sets and/or exercises (seconds); RBTS, rest between training sessions (hours); RBVJ, repeated bilateral vertical jumps; RT, resistance training; SSC, stretch-shortening cycle; Surf, surface type; T, tapering; TJ, total plyometric jumps. The symbol “>” denotes that only the minimum number of total plyometric jumps was able to be calculated from the data provided by the authors of the study. In addition, “s” denotes that the study incorporated sets of PJT drills prescribed in term of time (e.g., one set of 20 s of bouncing in place). The symbol “+” denotes that the study included sets of PJT drills prescribed as repetitions and time; TP, training period of the season; Type, type of PJT drill. When “Mix” is indicated, this involved a combination of 2 or more of the following jumping drills: vertical, horizontal, bilateral, unilateral, repeated, non-repeated, lateral, cyclic, sport-specific, slow stretch-shortening cycle, or fast stretch-shortening cycle; Wk, weeks of training*.

### Methodological Quality of the Included Studies

Among the included studies, 10 achieved a quality assessment of four-to-five points *(moderate quality)*, and eight achieved a quality assessment of 6–10 points *(high quality)*. A median PEDro score of 5 was noted across studies (Table 3).

**Table 3:** Physiotherapy Evidence Database (PEDro) scale ratings.

PEDro scale items^*^	**N^**°**^ 1**	**N^**°**^ 2**	**N^**°**^ 3**	**N^**°**^ 4**	**N^**°**^ 5**	**N^**°**^ 6**	**N^**°**^ 7**	**N^**°**^ 8**	**N^**°**^ 9**	**N^**°**^ 10**	**N^**°**^ 11**	**Total (from a possible maximal of 10)**
Amato et al. (2018)	1	1	0	1	0	0	0	1	1	0	1	5
Behrens et al. (2014)	1	1	0	1	0	0	0	1	0	1	1	5
Çımenlı et al. (2016)	1	1	0	1	0	0	0	1	1	1	1	6
Fathi et al. (2019)	1	1	0	1	0	0	0	1	1	1	1	6
Gjinovci et al. (2017)	1	1	0	0	0	0	0	0	1	1	1	4
Ho et al. (2019)	1	1	0	1	0	0	0	1	1	1	1	6
Idrizovic et al. (2018)	1	1	0	1	0	0	0	1	1	1	1	6
Kamalakkannan et al. (2011)	0	1	0	1	0	0	0	1	1	1	0	5
Kristicevic et al. (2016)	1	1	0	1	0	0	0	1	1	1	1	6
Maffiuletti et al. (2002)	1	1	0	1	0	0	0	1	1	1	1	6
Martel et al. (2005)	0	1	0	1	0	0	0	1	0	1	1	5
Newton et al. (1999)	1	1	0	1	0	0	0	1	1	1	1	6
Pereira et al. (2015)	0	1	0	1	0	0	0	1	0	1	1	5
Trajkovic et al. (2016)	1	1	0	1	0	0	0	1	1	1	1	6
Turgut et al. (2016)	1	1	0	1	0	0	0	1	1	1	0	5
Usman and Shenoy (2019)	1	1	0	1	0	0	0	1	1	1	0	5
Usman and Shenoy (2015)	0	1	0	1	0	0	0	1	0	1	1	5
Wang et al. (2020)	1	0	0	1	0	0	0	1	1	1	0	4
												Median score = 5

* *a detailed explanation for each PEDro scale item can be accessed at https://www.pedro.org.au/english/downloads/pedro-scale (access for this review: June 2, 2020, revisited on July 31, 2020)*.

### Meta-Analysis Results

The data used for meta-analyses are displayed in Table 4.

**Table 4:** Performances (mean, standard deviation and number of players) in physical fitness tests.

		**Experimental (pre-test)**			**Control (pre-test)**			**Experimental (pos-test)**			**Control (pos-test)**		
**References**	**Test**	**Mean**	**SD**	***n***	**Mean**	**SD**	***n***	**Mean**	**SD**	***n***	**Mean**	**SD**	***n***
**Linear sprint speed (seconds)**													
Fathi et al. (2019)	Sprint (10 m)	1.8	0.1	20	1.9	0.2	20	1.8	0.1	20	1.9	0.1	20
Fathi et al. (2019) rt	Sprint (10 m)	1.9	0.1	20				1.8	0.1	20			
Ho et al. (2019)	Sprint (10 m)	1.9	0.1	12	1.9	0.1	12	1.9	0.1	12	1.9	0.1	12
Idrizovic et al. (2018)	Sprint (20 m)	3.8	0.3	13	4.0	0.3	17	3.6	0.2	13	4.0	0.1	17
Gjinovci et al. (2017)	Sprint (20 m)	3.8	0.3	21	4.2	0.3	20	3.5	0.2	21	4.1	0.3	20
Kamalakkannan et al. (2011) loaded	Sprint (50 m)	7.6	0.4	12	7.6	0.4	12	6.8	0.5	12	7.5	0.3	12
Kamalakkannan et al. (2011) unloaded	Sprint (50 m)	7.6	0.4	12				7.3	0.5	12			
Turgut et al. (2016) weight-rope	Sprint (30 m)	5.8	0.3	8	5.8	0.2	8	5.4	0.3	8	5.8	0.2	8
Turgut et al. (2016) rope	Sprint (30 m)	5.4	0.3	9				5.7	0.1	9			
**Squat jump (cm)**													
Amato et al. (2018)	SJ	28.2	5.1	12	24.4	2.7	11	31.8	5.6	12	26.2	2.4	11
Fathi et al. (2019)	SJ	29.4	3.8	20	29.8	5.9	20	30.6	3.9	20	29.4	5.4	20
Fathi et al. (2019) rt	SJ	29.0	5.9	20				31.1	5.5	20			
Kristicevic et al. (2016)	SJ	21.8	4.2	28	24.3	4.1	26	24.3	3.5	28	24.8	4.1	26
Maffiuletti et al. (2002)	SJ	31.3	1.2	10	34.2	4.1	10	38.4	1.0	10	34.9	4.2	10
**Countermovement jump (cm)**													
Amato et al. (2018)	CMJ	29.5	6.8	12	26.4	4.5	11	34.1	6.3	12	29.5	4.0	11
Behrens et al. (2014)	CMJ	49.5	9.4	13	51.9	10.9	7	52.3	4.4	13	48.5	4.7	7
Fathi et al. (2019)	CMJ	32.6	6.8	20	32.2	6.0	20	33.7	6.8	20	32.4	5.8	20
Fathi et al. (2019) rt	CMJ	32.5	5.9	20				34.5	5.7	20			
Gjinovci et al. (2017)	CMJ	38.0	6.5	21	28.9	7.2	20	48.5	5.2	21	24.1	7.1	20
Idrizovic et al. (2018)	CMJ	42.2	6	13	41.7	4.3	17	49.5	7.0	13	45.1	5.1	17
Kristicevic et al. (2016)	CMJ	28.1	4.8	28	33.0	6.2	26	30.7	3.7	28	33.3	5.6	26
Maffiuletti et al. (2002)	CMJ	40.0	1.1	10	42.3	5.6	10	46.7	1.4	10	42.4	6.0	10
Pereira et al. (2015)	CMJ	26.9	4.5	10	25.0	3.7	10	32.3	9.0	10	25.8	3.7	10
Wang et al. (2020)	CMJ	67.0	3.8	10	66.9	4.1	10	69.3	3.9	10	66.8	3.4	10
**Drop jump (cm)**													
Amato et al. (2018)	DJ (40 cm)	27.8	8.0	12	24.5	4.7	11	30.7	7.3	12	24.8	3.7	11
Maffiuletti et al. (2002)	DJ (40 cm)	37.6	1.3	10	39.7	4.7	10	43.2	1.4	10	40.0	4.5	10
Newton et al. (1999)	DJ (30 cm)	0.57	0.1	8	0.6	0.1	8	0.6	0.1	8	0.6	0.1	8
**Spike jump (cm)**													
Newton et al. (1999)	Spike jump (3 steps)	78.0	6.2	8	80.4	6.2	8	83.0	7.2	8	80.5	7.4	8
Çımenlı et al. (2016) synthetic	Spike jump (1 step)	56.7	3.7	12	56.0	3.6	12	60.9	4.7	12	57.9	3.3	12
Çımenlı et al. (2016) wood	Spike jump (1 step)	59.2	5.8	12				65.9	6.7	12			
Maffiuletti et al. (2002)	Spike jump (3 steps)	56.9	1.2	10	53.0	4.8	10	63.6	1.3	10	54.4	4.8	10
**Countermovement jump with arm swing (cm)**													
Kamalakkannan et al. (2011) loaded	CMJ with arm swing	45.3	5.8	12	46.2	5.8	12	51.0	5.8	12	47.4	6.1	12
Kamalakkannan et al. (2011) unloaded	CMJ with arm swing	45.8	5.7	12				48.9	5.86	12			
Maffiuletti et al. (2002)	CMJ with arm swing	47.5	0.9	10	47.9	5.7	10	53.1	1.1	10	48.1	6	10
Martel et al. (2005)	CMJ with arm swing	33.4	4.7	10	31.9	5.3	9	37.1	4.5	10	33.2	4.7	9
Newton et al. (1999)	CMJ with arm swing	67.6	4.1	8	68.1	7.0	8	71.5	4.6	8	69.4	7.4	8

*CMJ, countermovement jump; DJ, drop jump; n, number of players/athletes measured; SD, standard deviation; SJ, squat jump*.

#### Linear Sprint Speed

**Figure 2:**
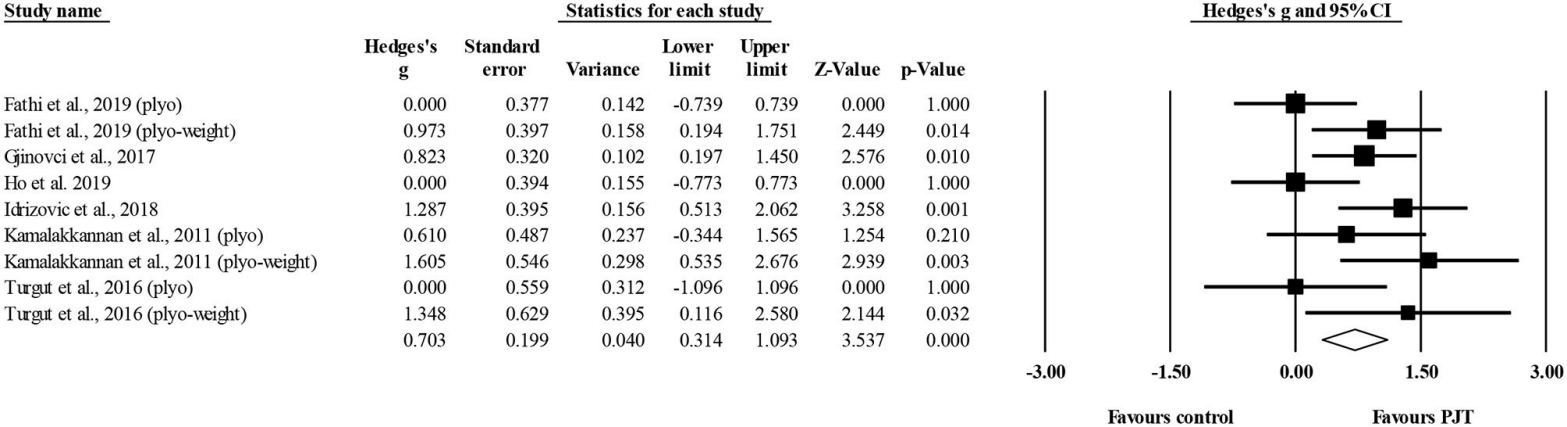
Forest plot of changes in linear sprint performance in volleyball players participating in plyometric jump training (PJT) compared to controls. Values shown are effect sizes (Hedges's g) with 95% confidence intervals (CI). The size of the plotted squares reflects the statistical weight of the study.

Six studies provided data for linear sprint performance, involving nine experimental and six control groups (pooled *n* = 216). Results showed a moderate effect of PJT on linear sprint performance (ES = 0.70; 95% CI = 0.31–1.09; *p* < 0.001; *I*^2^ = 46.1%; Egger's test *p* = 0.609; Figure 2).

#### Squat Jump

**Figure 3:**
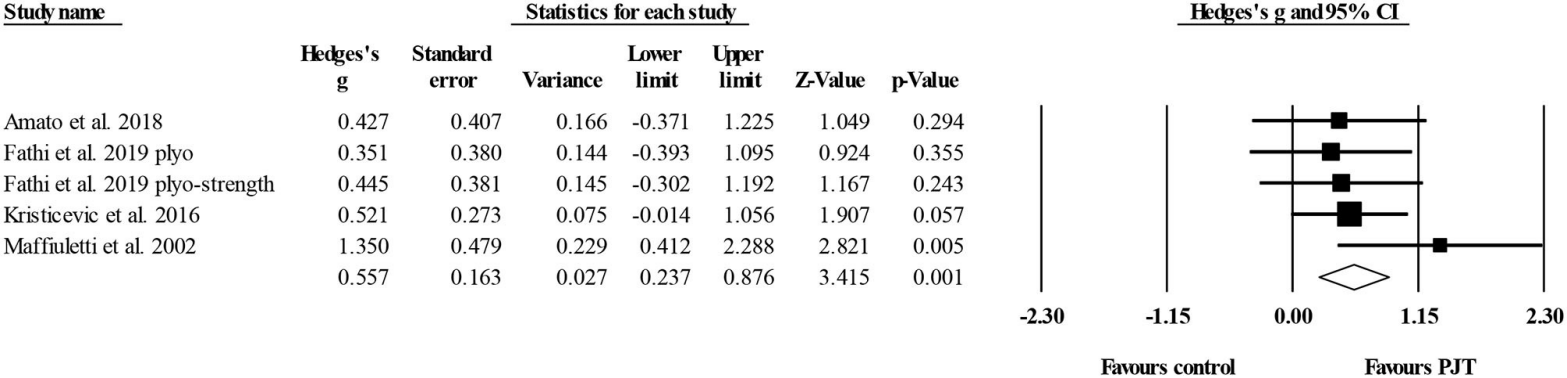
Forest plot of changes in squat jump performance in volleyball players participating in plyometric jump training (PJT) compared to controls. Values shown are effect sizes (Hedges's g) with 95% confidence intervals (CI). The size of the plotted squares reflects the statistical weight of the study.

Four studies provided data for squat jump performance, involving five experimental and four control groups (pooled *n* = 157). Findings indicated a small effect of PJT on squat jump performance (ES = 0.56; 95% CI = 0.24–0.88; *p* = 0.001; *I*^2^ = 0.0%; Egger's test *p* = 0.409; Figure 3).

#### Countermovement Jump

**Figure 4:**
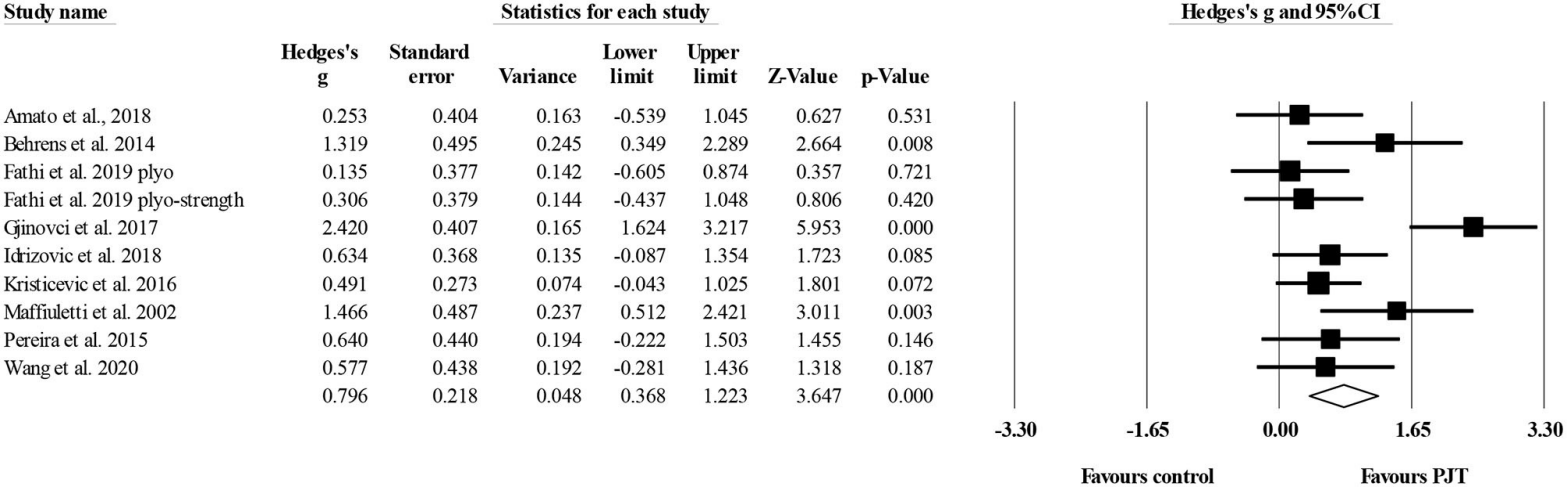
Forest plot of changes in countermovement jump performance in volleyball players participating in plyometric jump training (PJT) compared to controls. Values shown are effect sizes (Hedges's g) with 95% confidence intervals (CI). The size of the plotted squares reflects the statistical weight of the study.

Nine studies provided data for countermovement jump performance, involving 10 experimental and eight control groups (pooled *n* = 288). There was a moderate effect of PJT on countermovement jump performance (ES = 0.80; 95% CI = 0.37–1.22; *p* < 0.001; *I*^2^ = 66.8%; Egger's test *p* = 0.270; Figure 4).

#### Drop Jump

**Figure 5:**
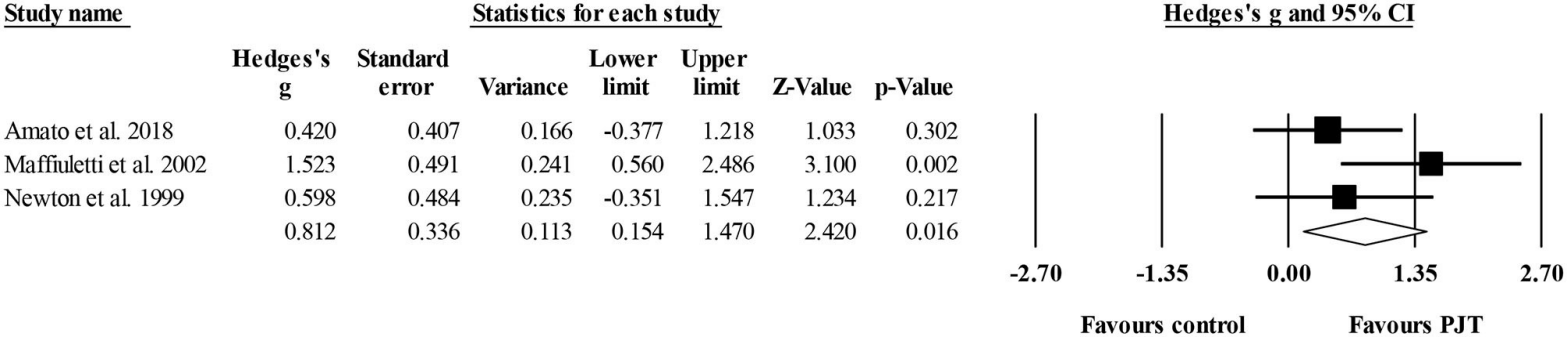
Forest plot of changes in drop jump performance in volleyball players participating in plyometric jump training (PJT) compared to controls. Values shown are effect sizes (Hedges's g) with 95% confidence intervals (CI). The size of the plotted squares reflects the statistical weight of the study.

Three studies provided data for drop jump performance, involving three experimental and three control groups (pooled *n* = 59). There was a moderate effect of PJT on drop jump performance (ES = 0.81; 95% CI = 0.15–1.47; *p* = 0.016; *I*^2^ = 37.6%; Egger's test *p* = 0.496; Figure 5).

#### Spike jump

**Figure 6:**
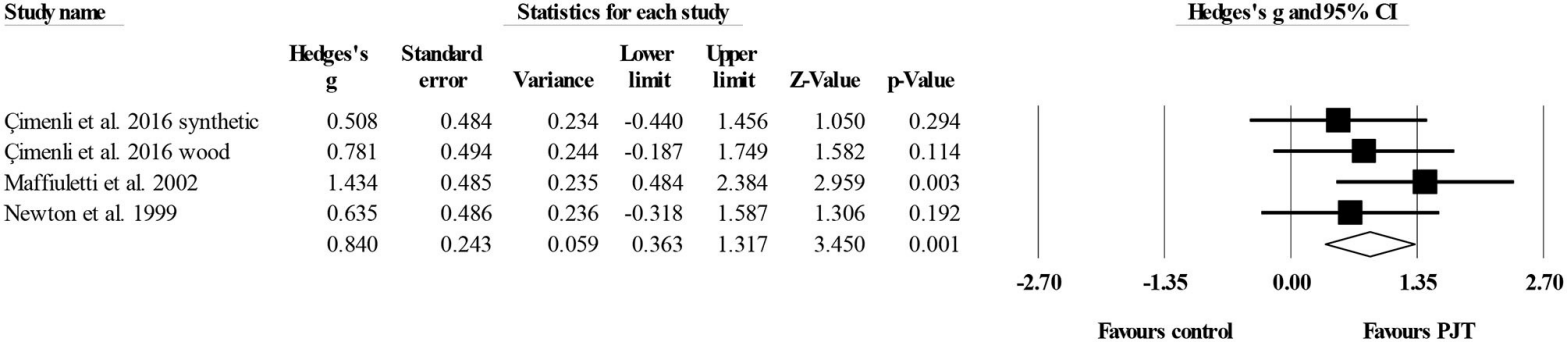
Forest plot of changes in spike jump performance in volleyball players participating in plyometric jump training (PJT) compared to controls. Values shown are effect sizes (Hedges's g) with 95% confidence intervals (CI). The size of the plotted squares reflects the statistical weight of the study.

Four studies provided data for spike jump performance, involving five experimental and four control groups (pooled *n* = 132). The Egger's test revealed a *p* = 0.048. After sensitivity analysis, the removal of one study (Trajkovic et al., 2016) allowed an Egger's test *p* ≥ *0.05*. As such, three studies with four experimental and three control groups were finally considered. There was a moderate effect of PJT on spike jump performance (ES = 0.84; 95% CI = 0.36–1.32; *p* = 0.001; *I*^2^ = 0.0%; Figure 6).

#### Countermovement Jump With Arm Swing

**Figure 7:**
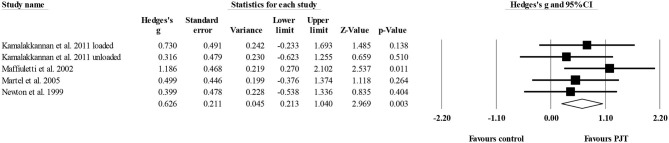
Forest plot of changes in countermovement jump with arm swing performance in volleyball players participating in plyometric jump training (PJT) compared to controls. Values shown are effect sizes (Hedges's g) with 95% confidence intervals (CI). The size of the plotted squares reflects the statistical weight of the study.

Six studies provided data for countermovement jump with arm swing, involving nine experimental and six control groups (pooled *n* = 301). The Egger's test revealed a *p* = 0.002. After sensitivity analysis, the removal of one study group publications (Usman and Shenoy, 2015, 2019) allowed an Egger's test *p* ≥ *0.05*. In the end, four studies with five experimental and four control groups were considered. There was a moderate effect of PJT on countermovement jump with arm swing performance (ES = 0.63; 95% CI = 0.21–1.04; *p* = 0.003; *I*^2^ = 0.0%; Figure 7).

### Additional Analyses

No significant sub-group differences (*p* = 0.758) were found between <8 weeks (and <16 total PJT sessions) (ES = 0.70; 95% CI = 0.35–1.05; within-group *I*^2^ = 15.7%, six study groups) and ≥8 weeks (and ≥16 total PJT sessions) (ES = 0.87; 95% CI = −0.12 to 1.85; within-group *I*^2^ = 85.6%, four study groups) of training. Similarly, no significant sub-group differences (*p* = 0.155) were found between <972 total jumps (ES = 1.14; 95% CI = 0.30–1.99; within-group *I*^2^ = 80.1%, five study groups) and ≥972 total jumps per programme (ES = 0.49; 95% CI = 0.17–0.80; within-group *I*^2^ = 0.0%, five study groups). Moreover, no significant sub-group differences (*p* = 0.422) were identified between amateur (ES = 0.62; 95% CI = 0.19–1.05; within-group *I*^2^ = 38.5%, six study groups) and professional volleyball players (ES = 1.01; 95% CI = 0.16–1.87; within-group *I*^2^ = 82.7%, four study groups). In addition, no significant sub-group differences (*p* = 0.598) were found between studies that evaluated the effects of single-mode PJT (ES = 0.87; 95% CI = 0.31–1.42; within-group *I*^2^ = 72.7%, seven study groups) compared with combined PJT including other training types (ES = 0.62; 95% CI = −0.09 to 1.34; within-group *I*^2^ = 55.4%, three study groups).

**Figure 8:**
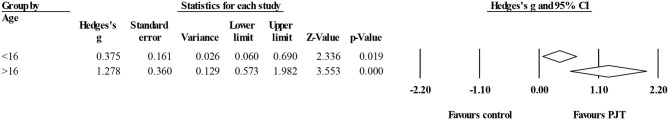
Forest plot of changes in countermovement jump performance in volleyball players (by years of age: <16 vs. ≥16) participating in plyometric jump training (PJT) compared to controls. Values shown are effect sizes (Hedges's g) with 95% confidence intervals (CI).

Nevertheless, significant sub-group differences (between-group *p* = 0.022) were observed between participants ≥16 years (ES = 1.28; 95% CI = 0.57–1.98; within-group *I*^2^ = 70.7%, five study groups) and <16 years (ES = 0.38; 95% CI = 0.06–0.69; within-group *I*^2^ = 0.0%, five study groups) (Figure 8).

Of note, the sub-analyses considered to assess the potential effects of eight moderator variables: programme duration (number of weeks), training frequency (number of sessions per week), total number of training sessions, and the total number of jumps, in addition to the sex, age, and the expertise level of the participants, and the comparison between studies that applied PJT-only and those that applied PJT combined with other training modality. In addition, our meta-analysis considered six main outcomes: linear sprint speed, squat jump, countermovement jump, drop jump, spike jump, and countermovement jump with arm swing. Therefore, 48 moderator analyses were potentially available. Nonetheless, due to a limited number of studies (i.e., <3 per moderator), only six moderator analyses were possible (as indicated above).

### Adverse Effects

None of the included studies reported soreness, pain, fatigue, injury, damage, or adverse health effects related to the applied PJT interventions, or related to games and other training contents. However, none of the studies considered adverse health effects as a primary or secondary outcome. Therefore, for most of the included studies, it is unclear whether no adverse health effects actually occurred or whether they were simply not reported.

## Discussion

This systematic review and meta-analysis examined the effects of PJT on measures of physical fitness in amateur and professional volleyball players. Eighteen controlled trials with 746 participants were included. Findings showed that PJT resulted in significant small-to-moderate improvements in measures of physical fitness in amateur and professional volleyball players. Our results indicated moderate enhancements in spike jump and CMJ with arm swing, both considered as volleyball-specific jump movements, following PJT. Due to the limited number of studies, moderator analyses were only possible for CMJ. Higher PJT-related improvements were observed in athletes ≥16 years old compared with those <16 years old, while similar moderate improvements were observed in both amateur and professional athletes.

### Primary Analyses

The main findings of this study showed beneficial effects of PJT on measures of physical fitness in volleyball players. Specifically, small-to-moderate enhancements on proxies of muscle power (squat jump, CMJ, CMJ with arm swing, drop jump, and spike jump) and linear sprint speed were observed, concurring with the wider strength and conditioning literature available for other sports (van de Hoef et al., 2019; Ramirez-Campillo et al., 2020a,e). A recent meta-analysis (Ramirez-Campillo et al., 2020b) revealed that PJT resulted in moderate improvements in CMJ height in volleyball players, regardless of players' age and sex. Our findings complement those of the aforementioned meta-analysis (Ramirez-Campillo et al., 2020b) and showed that, other than for CMJ height, PJT is also effective in enhancing sprint speed performance. In volleyball, a quick running approach before the jump is also related to a higher jump height (Wagner et al., 2009). More importantly, PJT induced moderate improvements on spike jump and CMJ with arm swing, considered as volleyball-specific jumps. In addition, our statistical calculation revealed only small enhancements in squat jump performances. As CMJ, CMJ with arm swing, drop jump, and spike jump are more dependent on the SSC compared to a squat jump, this may help explain the aforementioned findings. When performing an squat jump, there is no eccentric-concentric transition, limiting the use (if any) of elastic potential energy, decreasing the potentiation effect based on the SSC (Asmussen and Bonde-Petersen, 1974). In this sense, based on the principle of training specificity, greater improvements can be expected after PJT in activities that stress the SSC such as CMJ, CMJ with arm swing, drop jump, spike jump, and sprint speed compared with actions that do not involve the SSC (e.g., squat jump) (Bouguezzi et al., 2020a). Of note, Sattler et al. (2012) examined the interrelationships between generic jumps (e.g., CMJ, squat jump) and sport-specific jumps (e.g., block and spike [attack] jump) in elite male volleyball players. Factorial analyses resulted in one significant component indicating that all jump tests were inter-correlated. Accordingly, although speculative, it appears plausible to argue that PJT, via increment in vertical jump ability, can contribute to the improvement of players' performance during competition (Arnason et al., 2004; Sattler et al., 2015).

Improvements in jump performance can generally be attributed to factors such as enhanced motor unit recruitment, greater inter-muscular coordination, enhanced neural drive to agonist's muscles, and better utilization of the SSC (Markovic and Mikulic, 2010; Taube et al., 2012). With regards to training-induced enhancements in sprint speed, the optimal approach seems to incorporate greater horizontal acceleration (skipping, jumping with horizontal displacement) (de Villarreal et al., 2012; Ramirez-Campillo et al., 2015), particularly considering that forward acceleration is the most common sprint movement in volleyball (Polglaze and Dawson, 1992). Indeed, most (15 out of 18) of the included studies in our meta-analyses incorporated horizontal PJT drills (Table 2). Such a training approach may increase chances of gaining speed adaptations, considering the importance of horizontal force production and application for speed performance (Morin et al., 2012, 2015). In addition, gains in sprint performance may reflect adaptations such as an increased nerve conduction velocity, improved intra- and intermuscular coordination (Moritani, 1993; Markovic et al., 2007; Markovic and Mikulic, 2010; Wu et al., 2010), increased stiffness of the muscle-tendon complex (Markovic and Mikulic, 2010), and athletes' capacity to generate greater ground reaction forces and faster movement velocities (Morin et al., 2012), physiological and neuromechanically adaptations that may, potentially, transfer to other performance-determining skills in volleyball players.

### Results of Subgroup Analyses

In terms of the difference between participants ≥16 years old and <16 years old, the greater improvements in CMJ in the former is in line with previous studies (Moran et al., 2017a, 2018b) in which older youths improved more than younger youths, probably due to a higher number of exploitable pathways of adaptation in the former (neural and morphological) compared to their younger counterparts (neurological only) (Moran et al., 2017a, 2018b; Radnor et al., 2017). However, the aforementioned meta-analyses (Moran et al., 2017a, 2018b) focused on participants younger than 18 years, whereas in this meta-analysis older participants (>18 years) were included. Future studies may elucidate how maturity and/or training age may interact with PJT and physical fitness changes.

Of note, the moderator analyses revealed no difference between those PJT interventions which were <8 weeks in duration and included <16 total PJT sessions compared with those that lasted ≥8 weeks and incorporated ≥16 total PJT sessions. Similarly, no significant differences were found in CMJ improvements between PJT interventions with < ~1,000 total jumps compared to those with ≥972 total jumps per programme. Moreover, the improvement in CMJ after a PJT programme with a greater volume of jumps yielded only a small effect (ES = 0.47), whereas a moderate effect was noted with lower-volume PJT programmes (ES = 1.12). Additionally, greater volumes of PJT have been associated with increased injury risk, particularly in females (Brumitt et al., 2016, 2018). In this context, a previous study among physically active participants reported that either low (420 jumps), moderate (840 jumps), or high (1,680 jumps) jump volumes induced similar improvements in physical fitness, including jumping and sprinting (de Villarreal et al., 2008). Likewise, when a moderate PJT volume is realized across 8 weeks of training, there is evidence that a higher PJT frequency has no extra effects on male soccer players' physical fitness, including sprint-time performance, squat jump height, countermovement jump height, and drop jump height (Bouguezzi et al., 2020b). Findings from this meta-analysis are also in line with another study (Chaabene and Negra, 2017), which contrasted the effects of low vs. high PJT volume. After 8 weeks of training, similar effects were found for jump performance following low and high volume interventions in male soccer players. Therefore, it can be argued that a relatively low PJT volume could be effective to improve volleyball players' physical fitness, and may help them to devote more time to other key aspects of their preparation. Such findings may point toward the importance of the contents of PJT rather volume of training (Ramirez-Campillo et al., 2015; Bouguezzi et al., 2020a), an issue that deserves to be investigated in the future.

### Adverse Effects

The relative safety of PJT programmes has previously been reported (Markovic and Mikulic, 2010; Ramirez-Campillo et al., 2018a, 2020c). Moreover, when adequately programmed and supervised, PJT interventions may reduce the risk of sustaining injuries (Rossler et al., 2014, 2016). No intervention-related injuries were reported in the included studies. However, several included studies (Newton et al., 1999; Maffiuletti et al., 2002; Kamalakkannan et al., 2011; Behrens et al., 2014; Pereira et al., 2015; Usman and Shenoy, 2015, 2019; Çımenlı et al., 2016; Turgut et al., 2016; Gjinovci et al., 2017; Amato et al., 2018) did not provide information on adverse health effects following PJT. Relatedly, two studies concluded that PJT may lower the athlete's risk of sustaining injuries (Kristicevic et al., 2016; Trajkovic et al., 2016), although no data supported such a conclusion. One study reported no injury after PJT (Fathi et al., 2019) and another study (Martel et al., 2005) indicated no significant muscle soreness or injuries resulting from PJT. We have to acknowledge though that both studies did not report sufficient data to verify these statements. In addition, four studies reported safety considerations related to PJT programming in order to avoid excessive loads, fatigue and/or musculoskeletal complaints (Ho et al., 2016; Kristicevic et al., 2016; Idrizovic et al., 2018; Wang et al., 2020), although no data were reported for these outcomes. Overall, it seems that no adverse effects occurred following PJT interventions. However, due to the lack of reported information, these statements must be viewed with caution.

Indeed, although PJT seems safe for volleyball players, caution is needed when applying this type of training in poor-conditioned athletes with lower strength levels and an inability to decelerate their body mass during landing tasks. Moreover, a typical volleyball squad (n~12) performs ~120,000 jumps throughout a season (i.e., considering only the training sessions) (Garcia-de-Alcaraz et al., 2020). As this meta-analysis revealed similar jump performance improvements after PJT interventions with low (<1,000) vs. high (>1,000) total jumps, an effective approach to reduce potential adverse health effects due to excessive volume of plyometric load is to introduce a rather conservative PJT volume (e.g., ~120 jumps per week, distributed in two weekly training sessions). Additionally, taper strategies may also be of value (Ramirez-Campillo et al., 2020d). It appears possible that a reduction in PJT volume during the last stage of a PJT programme can reduce inflammation caused by overload-induced large eccentric loads (Choi, 2014; Fransz et al., 2018). Accordingly, a tapering strategy may help to avoid injuries and facilitate the processes of adaptation of the musculoskeletal system (Mujika, 2009).

### Future Lines of Research Inquiry

Of note, the majority of tests used to measure the effects of PJT addressed the vertical vector (i.e., jump with or without the movement of arms, with a running approach, etc.). Surprisingly, an insufficient number of studies (i.e., <3) incorporated tests that addressed the horizontal vector (i.e., agility behaviors or change-of-direction tests). This should constitute a future line of inquiry as volleyball performance is characterized by both, vertical and horizontal actions with quick displacements (block movements, defenses, attack after reception, etc.) (Sheppard et al., 2007; García de Alcaraz et al., 2017; Garcia-de-Alcaraz et al., 2020). In addition, measures of the effects of PJT (including upper-body drills) on a range of movements related to volleyball performance, including serve precision and velocity (Behringer et al., 2013; Fernandez-Fernandez et al., 2016), could constitute another pathway for a future studies.

Although all included studies in our meta-analysis were classified as being of moderate-to-high quality, none scored more than 6 points in the PEDro scale. Previous systematic reviews that focused on PJT (Johnson et al., 2011; Bedoya et al., 2015; Stojanović et al., 2017) also suggested that published studies in this area are generally of medium quality. This is likely due to the difficulties in conducting studies that include blinding of participants or therapists. Relatedly, a recent PJT scoping review (Ramirez-Campillo et al., 2020c) highlighted several methodological shortcomings from 420 analyzed studies, in particular an incomplete description of training intervention characteristics. Even though the included studies in this meta-analysis generally provided a clear description of the training intervention, some key elements, such as the recovery time between sets and repetitions, were reported only in two out of 16 studies. Future studies should strive for a more robust methodological approach.

In addition, our analyses revealed that PJT, when combined with other training types (e.g., resistance training), was not more effective than single-mode PJT to improve jump and sprint performances of volleyball players. However, such findings must be interpreted with caution. Admittedly, a limited number of studies was available for our analyses (Maffiuletti et al., 2002; Amato et al., 2018; Fathi et al., 2019; Usman and Shenoy, 2019). Further, recent meta-analyses suggested that complex training (e.g., combined resistance with PJT exercises) (Freitas et al., 2017; Cormier et al., 2020; Thapa et al., 2021) might favor meaningful physical fitness improvements in team sport athletes. Of note, among the four studies of this meta-analysis that applied combined PJT with other training types (Maffiuletti et al., 2002; Amato et al., 2018; Fathi et al., 2019; Usman and Shenoy, 2019), none followed complex training. Future studies may compare the effectiveness of single-mode PJT vs. complex training in volleyball players.

### Limitations

Some potential limitations of this review should be acknowledged. Additional analyses regarding PJT frequency, duration, total PJT sessions/jumps, expertise level/sex of athletes were not always possible as <3 studies were available for at least one of the moderators. Additionally, the dichotomisation of continuous data (e.g., ≥8 weeks compared to <8 weeks) with the median split technique could result in residual confounding and reduced statistical power (Altman and Royston, 2006). Furthermore, the programming parameters were calculated as single factors, irrespective of between-parameter interactions. For example, the effects of PJT interventions ≥8 weeks compared to <8 weeks on jump performance did not consider the intensity of the PJT drills. Regrettably, a multivariate random effects meta-regression was precluded due to the limited number of studies available reporting for a given outcome (*n* < 10) (Higgins et al., 2019). In addition, even though the included studies did not specify any adverse events associated with the PJT intervention, it is unclear if there was an attempt by the researchers to comprehensively record all possible adverse events. Therefore, future studies are encouraged to describe with more detailed data about possible injuries, pain, and/or any other potential adverse effects, as this would expand our knowledge on the safety of PJT. Further, none of the studies included in our meta-analysis clarified the overall training load for the experimental and control groups, including total jumps and training load during regular volleyball training and competitions, as well as training load associated with physical education classes or practice of other sports among youths. Therefore, it is possible that some of the observed differences between experimental and control groups arise from training-related factors other than PJT. Finally, although our meta-analysis followed internationally accepted guidelines (Liberati et al., 2009), we acknowledge that we did not follow an *a priori* registration.

### Practical Applications Derived From the Meta-Analysis

According to the results of our meta-analysis including males and females, as well as youth and adult volleyball amateur and professional players, PJT programs in combination with regular sport-specific training induced meaningful improvements in several physical fitness measures which are key for performance in volleyball, such as countermovement jump with arm swing and spike jump, in addition to sprinting over short distances.

From a periodization stand point, it might be argued that PJT should be initiated at an early stage of the athlete's career (i.e., younger athletes) to promote fundamental movement skills, progressing to latter stages of the athlete's development with greater intensity, specificity, and/or complexity (Lloyd et al., 2011). However, the mean duration of the included studies in our meta-analysis was ~8 weeks with a range of 5–16 weeks, precluding the application of the aforementioned long-term athlete development model. Moreover, the reported intensity of the included PJT programs was similar for youth and adult athletes. For example, among the five studies that reported maximal intensity (Table 1), two were conducted with adult athletes and three with young athletes. Further, eight of the included studies did not report specifics of the training period (e.g., in-season) in which PJT was applied. Four studies were conducted during in-season, five studies during pre-season and one study reported a period spanning from end of the pre-season to beginning of the in-season (Table 1). Furthermore, different progressive overload models were noted among the included studies, with four using a volume-based overload, another four studies using an intensity-based overload, one study reporting a technique-based overload, eight studies using a combination of volume- intensity- and technique-based overload, and one study did not use progressive overload at all (Table 1). Of note, most studies (*n* = 14) did not apply tapering. Therefore, from the available studies it is difficult to draw practical recommendations regarding PJT periodization in volleyball.

However, regarding the characteristics of effective PJT interventions, it seems that a training frequency of 1–3 sessions per week, over 8 weeks, is an adequate stimulus to boost physical fitness. Most studies incorporated some form of drop-like jump, although most studies included different types of jump drills into their programs. The total number of jumps varied greatly among the analyzed studies, with some studies including >4,000 jumps. Caution is warranted when high volume of PJT is prescribed. Indeed, a high volume of PJT may increase the injury risk (Brumitt et al., 2018). As a moderate volume of PJT may be as effective compared to a program with a greater volume among volleyball players (<972 vs. ≥972 total jumps), a moderate-volume of PJT is advised, particularly during initial stages of PJT, in those unexperienced with PJT, poor technical ability, and reduced ability to cope with the eccentric forces associated to jumping drills. Although our meta-analysis revealed similar improvements in countermovement jump height after single-mode PJT compared with combined PJT and other training types (e.g., RT), none of the included studies conducted complex training. A combination of resistance training with PJT drills may offer additional value to further improve volleyball players' performance (Freitas et al., 2017; Cormier et al., 2020; Thapa et al., 2021).

Although our results support the effectiveness of PJT to improve physical fitness of volleyball players, none of the included studies reported information related to the inter-individual responses to PJT, for instance according to the player's position (e.g., libero; setter; blocker). Considering the potential inter-individual variability in response to PJT among team-sport athletes (Meylan and Malatesta, 2009; Jiménez-Reyes et al., 2017; Ramirez-Campillo et al., 2018b) and that player's characteristics and demands during a game may vary according to their position in the field (García de Alcaraz et al., 2017; Garcia-de-Alcaraz et al., 2020), it can be argued that training-induced responses and adaptations to a given PJT stimulus may vary among players. Therefore, an individualized approach according to the player's position is warranted in future studies.

The included PJT studies reported no injuries. Indeed, PJT may be considered an integral part of neuromuscular training programs which focus on injury prevention (Rossler et al., 2014; ter Stege et al., 2014). However, a cautious approach is recommended, initially including moderate loads and adequate progression, particularly for those unexperienced with PJT and/or with an insufficient strength and conditioning base.

## Conclusion

This systematic review and meta-analysis indicated that PJT is safe (no reported injuries) and effective in improving measures of physical fitness (i.e., muscle power [squat jump, CMJ, drop jump, spike jump, and CMJ with arm swing], linear sprint speed) in amateur and professional volleyball players, including studies conducted with both male and females athletes. Particularly, PJT is effective in enhancing volleyball specific actions including spike jump and CMJ with arm swing. Results of subgroup analyses showed that PJT is more effective for CMJ improvement in volleyball players aged ≥16 years compared with younger players. Similar PJT-related effects were observed in amateur and professional players. Due to a limited number of studies (<3 per moderator variable), more research is needed to elucidate potentially moderating effects of age, sex, and expertise level following PJT on measures of physical fitness in volleyball players.

## Data Availability Statement

The original contributions presented in the study are included in the article/supplementary material, further inquiries can be directed to the corresponding author/s.

## Author Contributions

All authors made significant contributions, including preparation of the first draft of the manuscript, data collection, analysis of data, interpretation of data, provided meaningful revision and feedback, read, and approved the final manuscript.

## Conflict of Interest

The authors declare that the research was conducted in the absence of any commercial or financial relationships that could be construed as a potential conflict of interest.
